# Respiratory Function and Changes in Lung Epithelium Biomarkers after a Short-Training Intervention in Chlorinated vs. Ozone Indoor Pools

**DOI:** 10.1371/journal.pone.0068447

**Published:** 2013-07-12

**Authors:** Álvaro Fernández-Luna, Leonor Gallardo, María Plaza-Carmona, Jorge García-Unanue, Javier Sánchez-Sánchez, José Luis Felipe, Pablo Burillo, Ignacio Ara

**Affiliations:** 1 School of Sports Science. European University of Madrid, Madrid, Spain; 2 IGOID Research Group, University of Castilla-La Mancha, Toledo, Spain; 3 Sports Science Institute. Camilo José Cela University, Madrid, Spain; 4 GENUD (Growth, Exercise, NUtrition and Development) Toledo Research Group, University of Castilla-La Mancha, Toledo, Spain; National Institutes of Health (NIH), United States of America

## Abstract

**Background:**

Swimming in indoor pools treated with combined chemical treatments (e.g. ozone) may reduce direct exposure to disinfection byproducts and thus have less negative effects on respiratory function compared to chlorinated pools. The aim of this study is to analyze the effects of a short-term training intervention on respiratory function and lung epithelial damage in adults exercising in indoor swimming pool waters treated with different disinfection methods (chlorine vs. ozone with bromine).

**Methods:**

Lung permeability biomakers [surfactant protein D (SP-D) and Clara cell secretory protein (CC16) in plasma] and forced expiratory volumes and flow (FEV1, FVC and FEF_25–75_) were measured in 39 healthy adults. Thirteen participants swam during 20 sessions in a chlorinated pool (CP), 13 performed and equivolumic intervention in an ozone pool (OP) and 13 were included in a control group (CG) without exposition.

**Results:**

Median plasma CC16 levels increased in CP swimmers (4.27±3.29 and 6.62±5.51 µg/L, p = 0.01, pre and post intervention respectively) while no significant changes in OP and CG participants were found. No significant changes in median plasma SP-D levels were found in any of the groups after the training period. FVC increased in OP (4.26±0.86 and 4.43±0.92 L, p<0.01) and CP swimmers (4.25±0.86 and 4.35±0.85 L, p<0.01). FEV1 only increased in OP swimmers (3.50±0.65 and 3.59±0.67, p = 0.02) and FEF_25–75_ decreased in CP swimmers (3.70±0.87 and 3.37±0.67, p = 0.02).

**Conclusion:**

Despite lung function being similar in both groups, a higher lung permeability in CP compared to OP swimmers was found after a short-term swimming program. Combined chemical treatments for swimming pools such as ozone seem to have less impact on lung epithelial of swimmers compared to chlorinated treated pools.

## Introduction

Nowadays swimming has become one of the most popular sports in western societies, mainly due to the positive physical and psychological health benefits associated to its regular practice, both in healthy and diseased populations (i.e. patients suffering chronic respiratory problems, degenerative neuromuscular disease and/or obese subjects among others) [1].

On the other hand, the practice of swimming in indoor pools has also generated interest from the medical perspective because of the possible negative health effects caused by the direct exposure to chemicals and disinfection byproducts (DBPs) generated by water disinfectants [2], [3]. Within these elements, chloramines and trihalomethane [4], have been identified as irritant products [5]. Recent case studies, have observed that the accidental increase of chlorine values above the established levels for indoor water pools is associated with adverse health effects in swimmers and swimming pool workers (lifeguards and instructors) including throat irritation, shortness of breath, wheezing, coughing and decrease in forced expiratory volumes [6], [7]. In fact, chronic exposure to DBPs and chlorine has been associated with eye, throat, skin and nasal irritation [8]–[10]. Additionally, the relationship between swimming practice and the risk of asthma and/or allergy has been suggested but not confirmed in epidemiological studies [11]–[13]. An alternative to reduce the potential harmful effects of chlorine and DBPs exposure is the use of complementary methods to chlorination disinfectants such as ozone and ultraviolet (UV) lamps. A significant decrease of chlorine and DBPs in installations with these chemical treatments were found [14], [15].

Several blood biomarkers related to the biological mechanisms behind respiratory problems have been recently applied for the study of this issue [7]. The lung surfactant protein named Clara Cell protein 16 (CC16) and the surfactant protein D (SP-D) secreted by the lung epithelium has been used as indicators to detect short-term changes in lung epithelial integrity [16]–[18] that can produce inflammation of the airways, increased sensibility and allergic diseases, produced by the high permeability of the epithelial barrier [19]. Additionally, forced expiratory volumes have also been used in numerous studies in swimmers, not just as a performance indicator [20] but also in order to observe changes in volumes as a symptom of changes in lung function [21]–[22]. Thus, the aim of the present study is to analyze the effects of a short-term training intervention on respiratory function and lung epithelial damage in adults exercising in swimming pool waters treated with different disinfection methods (chlorine vs. ozone with bromine).

## Materials and Methods

### Subjects

Participants were recruited through advertisement in the university and sports facilities in Toledo (Spain). Participants were fully informed of the nature and the possible risks associated with the study before they volunteered to participate and after they signed an informed consent. Initially, participants were recruited into three groups: chlorinated indoor pool (CP), ozone indoor pool (OP) and control (CG). A priori power analysis based on a medium effect size of 0.30 on the CC16 and SPD values revealed that eight participants were needed per group to reach a statistical power of 0.95 (G*Power 3.1.7 for Windows) [23]. Significance level (α = 0.05) and statistical power (1–β = 0.80) were set as initial values. The model effect size (dz = 0.833) was calculated from our previous data. Considering a dropout rate of 20% and aiming to increase the statistical power of the results, a number of 13 subjects per group were finally recruited (n = 39).

The study was approved by the Clinical Research Ethical Committee (CEIC) (13/10) of Castilla-La Mancha University and the experiments conformed to The Declaration of Helsinki. The exclusion criteria for all the groups included current smokers and those who suffer from asthma, chronic obstructive pulmonary disease or allergy. None of the participants were on medication at the time of the study.

### Study Design

Participants reported to the laboratory on 2 days over a 3-months period. All participants were instructed not to visit any indoor pool (including spas) for at least a week prior to the start of the study and pool participants were instructed to engage in its last training session the day before the last test day, and also to consume their normal diet. On each of the experimental days, participants presented themselves after fasting overnight and, after 15 min of rest a venous blood sample was obtained. Finally, a pulmonary function test was used to assess the lung function forced expiratory volume in one second (FEV1) and forced vital capacity (FVC) using a portable spirometer (Spirobank II, Medical International Research slr, Rome, Italy), following standard recommendations [24]. FVE1 volumes and FVC were expressed in liters. Some additional questions on current and former swimming and lifestyle habits (former smokers) were asked. On day 2, subjects from swimming groups were asked to answer a health survey about frequency of health complaints during training (i.e. eye irritation outside the pool, eye irritation in the pool, skin irritation, skin dryness, cough, throat irritation, breathing difficulties and otitis) using a likert scale of 1–7.

### Pools Characteristics

Water and environment quality variables as free and combined chlorine, bromine, pH, room and water temperature were evaluated in both swimming pools during the training program was performed. Both swimming pools are in compliance with the Spanish law of quality of water in swimming pools, making one filter backwashing per day and a complete fresh water replacement once per year. The researchers evaluated the water parameters once a week in each swimming pool. To asses these parameters a portable photometer (Logivond Water Testing, Tintometer GmbH, Dortmund) and a portable thermometer (Digital thermometer, Tfa Dostmann, Wertheim) were used.

### Training Program

The swimming training program consisted in 2–3 non-consecutive sessions per week for three months (20 sessions). The aim of the training was to improve the swimming technique styles. The duration of each session was 50 minutes with a daily training volume of 500±300 meters. The sessions took place in the afternoon between 19∶30 and 22∶00. The total number of hours of exposure for both the CP and the OP groups were 1000 minutes. The subjects from control group (CG) were non-training persons and they did not make any exercise during the study period.

### Analytical Procedures

For analysis of the plasma concentration of protein CC16 and SP-D, 5 ml of venous blood was collected of each participant and was stored in tubes containing anticoagulant. The samples were centrifuged at 4000 rpm for 10 minutes and the plasma from each sample was circulated respectively into eppendorf tubes to be stored at −80°C during three months. The protein concentration was analyzed by ELISA kits (Biovendor Laboratorní Medicine, Modrice, Czech Republic). The coefficients of variation intra-and inter-assay were 2.0% to 2.5% in both cases for the plasma SP-D and 4.0% to 5.0% for CC16. The minimum concentration in plasma protein was set at 0.2 µg/mL for SP-D and 20 µg/mL for CC16 (Biovendor Laboratorní Medicine). The levels obtained were expressed in micrograms per liter of plasma (µg/L).

### Statistical Analysis

Results are presented as mean ± SD, if not otherwise stated. A repeated-measure two ways ANOVA test was performed comparing values of CC16 and SP-D in plasma and forced expiratory values, before and after three months of training in chlorinated pool swimmers, ozone pool swimmers and control group. In the nonparametric data, Mann-Whitney test was used to compare the results of the health survey in CP vs. OP group. P<0.05 was used as the level of significance. Statistical analysis was performed using SPSS package v 19.0.0 (IBM, Chicago).

## Results

Table 1 summarizes general characteristics of the participants. All groups had comparable age. Both CP and OP training groups had similar swimming experience. No allergy symptoms were present in any of the participants (Table 1).

**Table 1:** Clinical characteristics of study participants (n = 39).

	All (n = 39)	Chlorine pool group(n = 13)	Ozone pool group(n = 13)	Control group (n = 13)
sex (M/F)	17/22	5/8	5/8	7/6
Age (y) (SD)	34.1 (7.4)	33.5 (7.7)	37.1 (5.37)	31.7 (8.4)
swimming experience (y) (SD)	–	5.1 (6.0)	8.0 (7.8)	–
former smokers	9	4	4	1
Allergy symptoms	–	–	–	–

The mean values of chemical concentration in water and temperature assessed in the chlorinated pool were: free chlorine in water: 1.1±0.3 mg/L, combined chlorine: 0.4±0.1 mg/L, pH: 7.4±0.2, room temperature: 27.8±1.5°C, water temperature: 25.5±0.3°C. Regarding the ozone pool, results were: total bromine, 1.8±0.3 mg/L, pH, 7.5±0.2, room temperature 28±0.8°C and water temperature 25.9±0.2°C.

As depicted in Table 2, values of perceived health problems were similar between groups in the likert scale with a frequency between 1–7, except for eye irritation that was higher in CP group compared to OP (p<0.05).

**Table 2:** Frequency of health complaints reported by swimmers (n = 26) during training (frequency measured by likert scale 1–7).

Symptoms	Chlorine pool group (CP) n = 13	Ozone pool group (OP) n = 13	*p* value
Eye irritation outside the pool	2.1 (1.5)	1.4 (0.5)	0.204
Eye irritation in the pool	3.1 (2.3)	1.9 (1.3)	**0.026**
Skin irritation	1.6 (0.9)	1.5 (0.6)	0.724
Skin dryness	3.4 (1.8)	2.8 (1.6)	0.656
Cough	1.3 (0.6)	1.4 (0.6)	0.762
Throat irritation	1.4 (0.7)	1.5 (0.7)	0.762
Breathing difficulties	1.2 (0.6)	1.1 (0.3)	0.724
Otitis	1.4 (0.7)	1.8 (1.4)	0.801

Data are expressed as mean (SD).

Plasma levels of SP-D and CC16 were similar in CP and OP groups before training program. SP-D plasma concentration was not significantly modified after the training period in neither CP, OP or C groups. However, plasma concentration of CC16 increased significantly in CP group (p<0.01) (Table 3).

**Table 3:** Changes in serum proteins levels CC16 and SP-D, and forced expiratory volumes: FEV1, FVC and FEF 25–75 before (PRE) and after (POST) a swimming program in adults (n = 39).

	Chlorine pool group (n = 13)			Ozone pool group (n = 13)			Control group (n = 13)		
	PRE mean (SD)	POST mean (SD)	*p* value	PRE mean (SD)	POST mean (SD)	*p* value	PRE mean (SD)	POST mean (SD)	*p* value
Serum levels									
CC16 (µg/L)	4.27 (3.29)	6.62 (5.15)	0.010	4.33 (2.28)	5.01 (2.99)	0.093	3.61 (1.48)	3.68 (1.35)	0.847
SP-D (µg/L)	98.51 (80.52)	97.73 (69.54)	0.923	101.23 (69.41)	102.08 (51.58)	0.954	113.39 (94.91)	103.66 (65.21)	0.354
Forced expiratory volumes									
FEV1(L)	3.56 (0.75)	3.51 (0.72)	0.102	3.50 (0.65)	3.59 (0.67)	0.025	4.00 (1.08)	4.09 (1.07)	0.199
FVC (L)	4.25 (0.86)	4.35 (0.85)	0.003	4.26 (0.86)	4.43 (0.92)	0.007	4.85 (1.43)	4.93 (1.44)	0.223
FEF 25–75 (L/s)	3.70 (0.87)	3.37 (0.67)	0.024	3.61 (0.76)	3.67 (0.73)	0.630	4.09 (1.01)	4.09 (0.81)	0.990

Forced expiratory values are shown in Table 3. FEV1 and FVC were similar in CP and OP groups before training program. FVE1 value did not change significantly after training program in CP group, however a significant increase in FVC value (p<0.01) was observed. OP group significantly improved in both expiratory volumes after training program, (both p<0.05). FEF_25–75_ decreased significantly in CP group (p<0.05). Forced expiratory volumes did not change in the CG group after the three month period.

## Discussion

Although a swimming program consisting of 20 one-hour sessions resulted in a similar increase in respiratory function in adults training in both chlorinated and ozone treated pools, the present investigation shows that swimming in a chlorine treated pool is associated with a significant increase in basal concentration of CC16 while no significant changes are observed in ozone pool swimmers. Furthermore, higher frequencies of eye irritation problems were also reported by chlorinated pool users.

**Figure 1:**
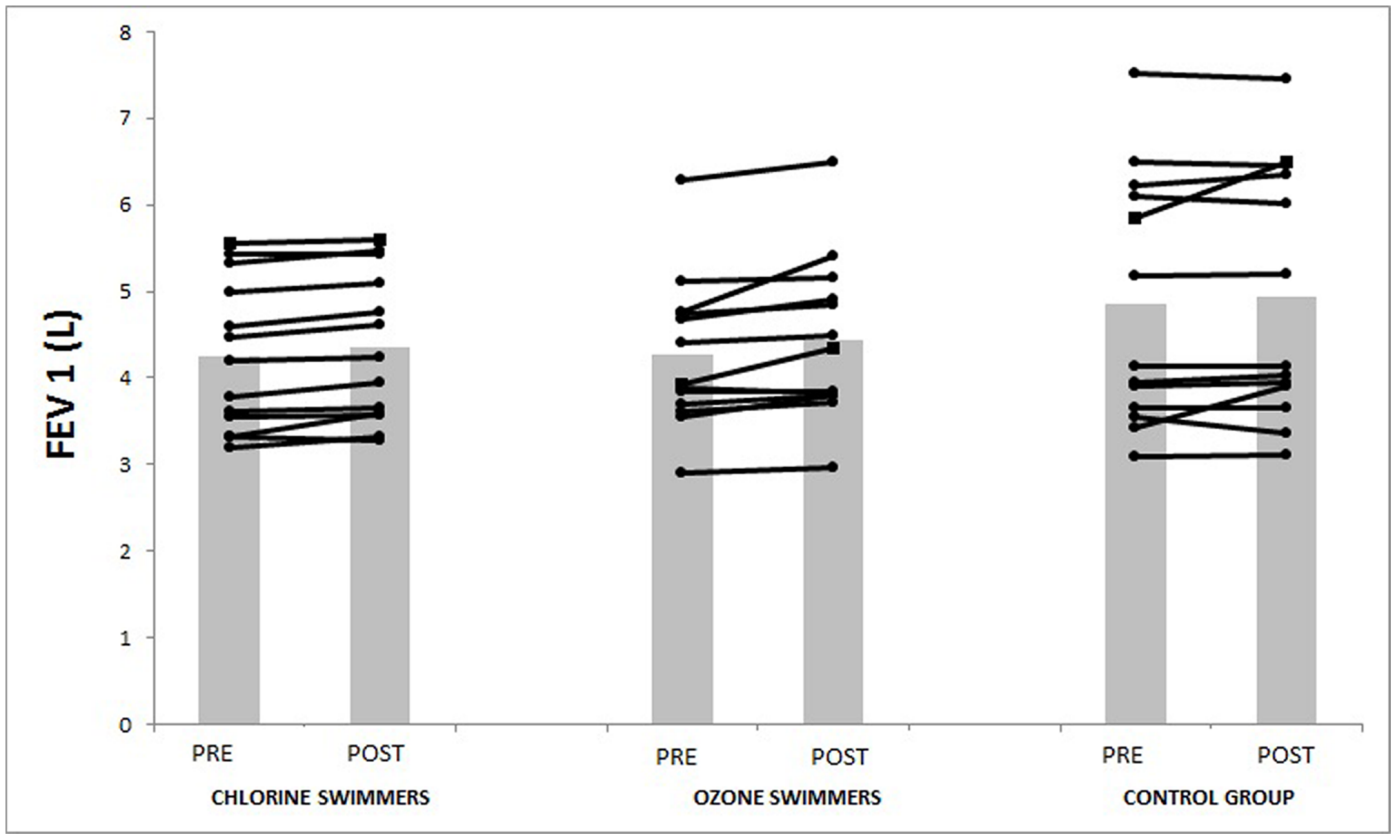
Changes in FEV1 pre and post intervention.

**Figure 2:**
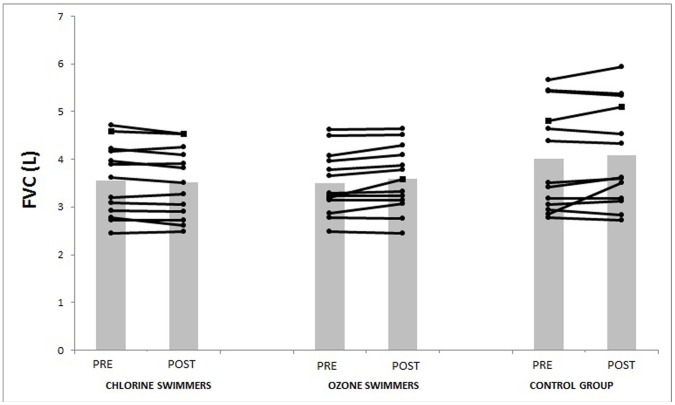
Changes in FVC pre and post intervention.

In adults, training programs have positive effects on lung function as showed by increased expiratory volumes [25]. Accordingly, in the present study the ozone pool swimmers improved in the two expiratory volumes analyzed (FVE1 and FVC) (Figure 1 and 2) and chlorinated pool swimmers improved in FEV1 (Figure 1). However, other authors require a 12–15% increase in FEV1 and/or FVC as necessary to define a meaningful response [26]. In our case the short exposure to the chemicals or the low intensity level of the physical training could explain the non-meaningful changes.

Moreover, although an exposure of chlorine gas caused by an accident could produce significant decreases in lung volumes [6], [7], in our study we can rule out this possibility as values of chemicals in water and temperature in both swimming pools were evaluated and proved to be among law ranges (Free Chlorine: 0.4–1.5 mg/L; Combined chlorine: <0,6 mg/L, Bromine: 1–3 mg/L; pH: 7–8; water temperature: <28°C; and room temperature: <30°C).

Additionally, an increase of the forced expiratory flow FEF_25–75_ has been observed in adults after a swimming program similar to the one that was used in our study [25]. But in our case we did not find an increase of FEF_25–75_ in OP and CG group, but a significant decrease was found in the CP group after the training period. The decrease of FEF_25–75_ has been associated with a damage in the smaller airways caused by the exposition to chemical compounds such as ambient ozone in adults [27]. As a consequence, we need further studies to assess if the chlorine in swimming pools affects the smaller airways.

However, prolonged exposure to DBPs in indoor pools has been suggested to be related to various health issues mainly at the skin, eye, throat and ears sites [8]–[10]. The fact that skin dryness had a high value in the likert scale is consistent with previous studies of perceived health problems for swimmers [28] and swimming pool workers [29]. The later being especially noted during winter months (our study was performed between January-March) and is normally caused by a combination of the dilution of natural sebum and by the osmotic gradient produced when the body is immersed in water, drawing hydration from the outer skin layers [8]. Nevertheless, only eye irritation was perceived more often in the chlorinated pool compared to the ozone pool and this may be due to the increased generation of DBPs in the chlorinated pool while the ozone system is known for removing chloramines and trihalomethanes during the disinfection process [15].

More recently, the use of lung epithelial damage serum biomarkers has been used in order to noninvasively study the effects of disinfection products on human health. CC16 and SP-D proteins are secreted into the airways and alveoli where they exert their main functions and their levels are detectable in blood and become elevated in lung injury, supporting the potential use of these as non-invasive biomarkers of inflammation, injury, and epithelial integrity of the lung lining surfaces [30].

Changes in serum CC16 level has been previously described in other studies after a single swimming session in a chlorinated indoor pool [19], [21], [22] but not in ozone treated pools. In our study, the absence of changes in this marker provides further evidence that a 20 hour-swimming program performed in an ozone treated pool does not acutely affect the lung epithelium. Thus, since all participants (chlorine and ozone pool swimmers) performed equivolumic training we can state that ozone pools might produce a lower impact in the lung permeability of swimmers after a short-term training period. This is in agreement with the well-known relationship between respiratory symptoms and the presence of DBPs in water and environment, which is higher in pools with chlorinated chemical treatments in respect to pools using ozone [15] but as an important limitation the concentration of DBPs was not measured in this study. Other studies have associated the increase of CC16 in the serum and urine of competitive swimmers with the intensity of physical activity, due to increased hyperventilation during intense exercise. [19], [21], [22], [31]. However, in our case, physical activity was not intense, since our objective was mainly to improve the technique of swimming styles and thus no intense exercises were included. It has been previously shown that high exposure of chlorine due to failure in security systems of the indoor swimming pools, results in greatly increased CC16 levels after 3–5 hours [32]. In our study post intervention measurements were made after a three months training intervention, the following day of the swimmers last training session.

In accordance with the present results, Font-Ribera et al. [19], did not observe any change in basal lung surfactant protein SP-D in plasma of swimmers probably due to the higher molecular weight of SP-D (130 KDa) [18] compared to CC16 (16 KDa) [17] that it would not permit the passive diffusion of the molecule through the epithelium barrier. In other studies, differences in serum concentrations of other surfactant proteins (SP-A, and SP-B) were found after a short exposure in a chlorinated swimming pool in recreational and trained swimmers, but not in a pool with copper-silver disinfection method [22], [23]. On the contrary, a significant increase in serum SP-A and SP-B concentrations in adults after one hour of pool side presence without swimming was previously found [21]despite no significant differences being found in serum SP-D levels between children who swan in a chlorinated pool during lactation and children who did not [33]. Moreover, there are other suitable biomarkers (i.e. cytokines) not included in the present study that could also add more information in relation to lung function [19].

In summary, very few studies have assessed the health consequences of similar swimming training programs performed in differently treated indoor pools using respiratory function assessment and serum biomarkers. The main finding of our study was that a significant increase of serum concentration of CC16, combined with an increased eye irritation was observed in chlorinated pools compared to ozone treated indoor pools after a short-term training program.
